# Wandering Spleen: A Rare Case From the Emergency Department

**DOI:** 10.7759/cureus.33246

**Published:** 2023-01-02

**Authors:** Mahdi Jawad, Mohamed H Yusuf, Kaltham A Al Doaibel, Fatema M Nesaif, Ahlam S Alharbi

**Affiliations:** 1 Radiology, Qatif Central Hospital, Qatif, SAU; 2 General Practice, Qatif Central Hospital, Qatif, SAU; 3 General Practice, Royal College of Surgeons in Ireland, Dublin, IRL; 4 Family Medicine, Primary Health Care Center, Riyadh, SAU

**Keywords:** case report, laparotomy, computed tomography, acute abdominal pain, wandering spleen

## Abstract

The spleen is typically located in the left upper quadrant and is held in position by the suspensory ligaments, which include the gastrosplenic ligament, the splenorenal ligament, and the phrenicocolic ligament. Abnormalities within these ligaments result in the mobility of the spleen, so it may be located in the pelvis or iliac region, which is termed a wandering spleen. We present a case of a middle-aged man who presented to the emergency department with generalized abdominal pain and diffuse guarding and tenderness. The patient had a previous history of peptic ulcer disease and multiple emergency department visits for gastritis. Given the assumed diagnosis of perforated viscus, the patient underwent a computed tomography scan that demonstrated the absence of the spleen in its usual location and showed an ectopic pelvic spleen. The patient underwent successful surgical treatment with splenopexy. The wandering spleen is a rare medical condition that presents a clinical diagnostic challenge and requires a high index of suspicion. Despite its rarity, the wandering spleen should be considered in patients with recurrent abdominal pain.

## Introduction

The spleen is normally located in the left hypochondriac region of the abdomen. It is primarily held in position by the suspensory ligaments, including the gastrosplenic ligament, the splenorenal ligament, and the phrenicocolic ligament. The absence or laxity of these ligaments leads to the migration of the spleen from its normal location to the pelvic region [1]. This condition is referred to as a wandering spleen, which is a rare condition. It is estimated that the incidence of wandering spleen is 0.2%, with fewer than 500 cases reported in the literature [2]. The wandering spleen may be due to congenital or acquired laxity of the ligaments. Due to hormonal factors, this condition is more common among women, particularly in multiparous women. Here, we present a case of a wandering spleen in a middle-aged man who presented with intermittent abdominal pain and underwent a successful splenopexy procedure. The clinical manifestations of the wandering spleen are variable, as it may be asymptomatic or present with acute abdomen due to splenic torsion [3].

## Case presentation

We present a case of a 42-year-old man who presented to the emergency department complaining of abdominal pain for one day. He reported that the pain was generalized and had a sudden onset. The pain was sharp in nature with no radiations. It was associated with nausea and vomiting. However, the pain was not related to food intake. The patient could not identify any aggravating factors. The pain was partially alleviated by the use of nonsteroidal anti-inflammatory drugs. He rated the pain as 7 out of 10 on a 10-point severity scale. The patient reported similar, but milder, episodes of such pain over the past six months that had spontaneous resolution. The past medical history of the patient was remarkable for hypertension, diabetes mellitus, and peptic ulcer disease. The patient did not undergo any previous surgery. The patient had multiple visits to the emergency department over the last five years for intermittent abdominal pain that was clinically diagnosed as gastritis. The medication history included metformin 1000 mg, amlodipine 5 mg, and omeprazole 40 mg daily. The family history was remarkable for colon cancer in his father who was diagnosed at 50 years of age.

On physical examination, the patient appeared in severe pain. There were no signs of respiratory distress. The vital signs included a respiratory rate of 15 breaths/minute, pulse rate of 110 beats/minute, blood pressure of 136/90 mmHg, and a temperature of 37.1°C (measured by oral route). Abdominal examination revealed a distended abdomen with generalized tenderness. The patient had diffuse guarding and rigidity, suggesting peritonitis. The bowel sounds had normal intensity and quality. Examination of the cardiac and respiratory systems was normal. The laboratory findings showed no significant abnormalities. Considering the patient's history of peptic ulcer disease, the provisional diagnosis was a perforated peptic ulcer disease. Hence, the patient was referred to have a computed tomography scan of the abdomen.

**Figure 1:**
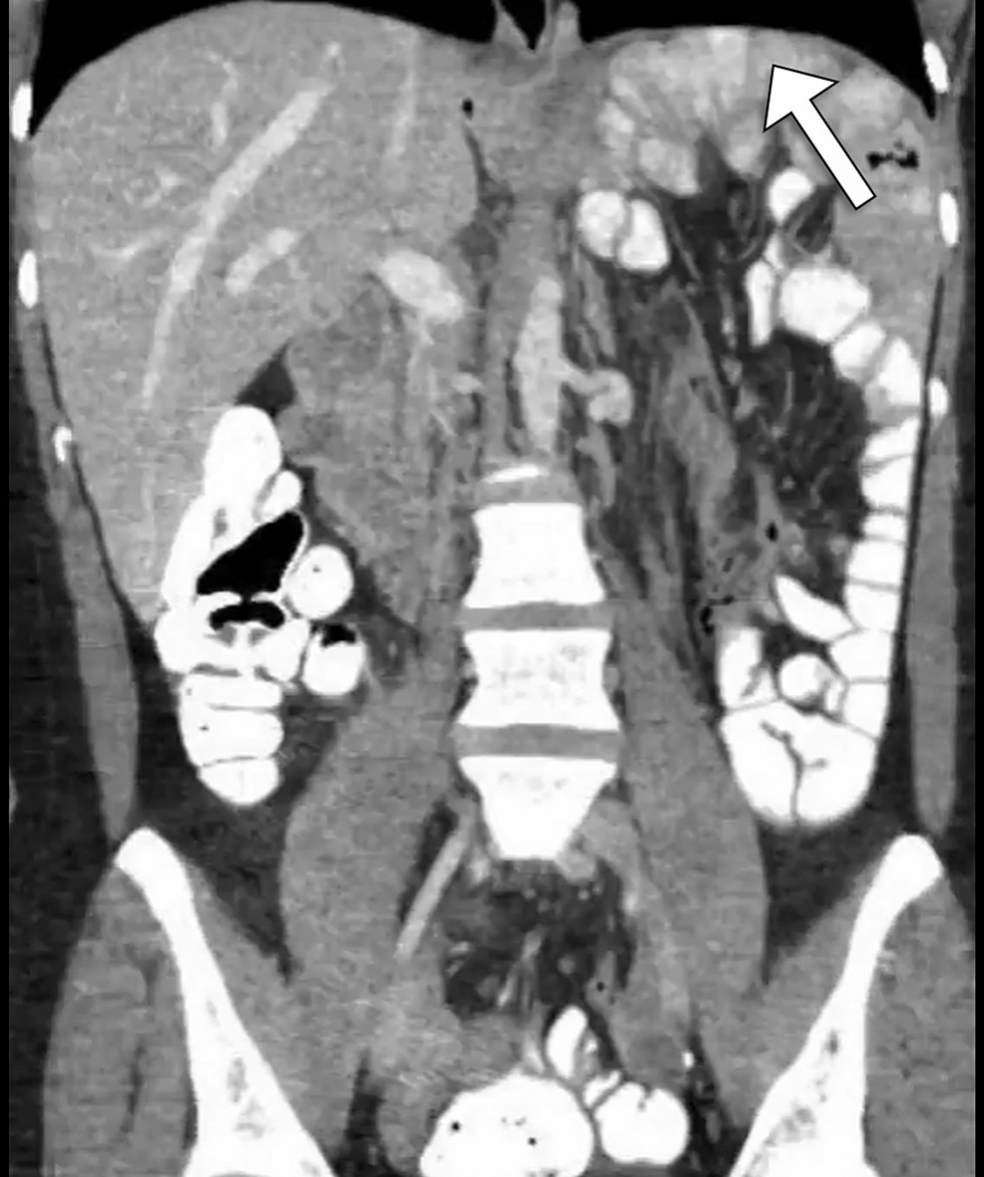
Coronal CT image of the abdomen shows the absence of the spleen in the upper quadrant of the abdomen (arrow).

**Figure 2:**
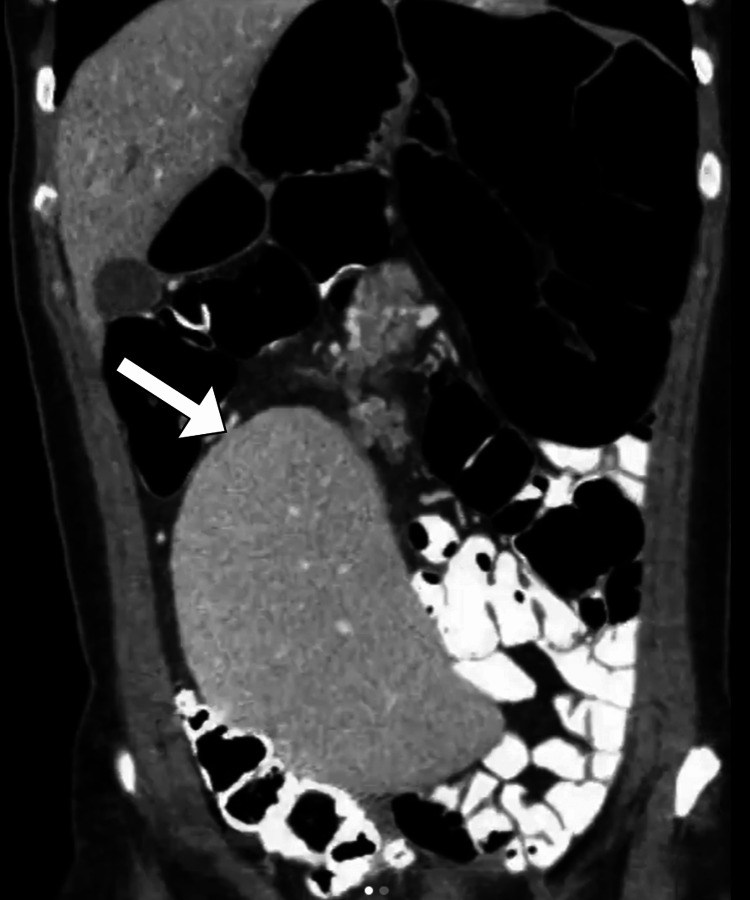
Coronal CT image of the abdomen shows a well-defined mass in the right hemiabdomen and pelvis (arrow) with a similar density of splenic tissue.

The computed tomography scan was performed after oral and intravenous contrast administration. The scan revealed the absence of the spleen in the expected normal location and showed a homogeneous pelvic mass of similar attenuation to that of the normal spleen. There were no hypodense lesions within the mass to suggest infarction. The supplying artery was patent. Such findings represented the diagnosis of a wandering spleen (Figures 1, 2).

Subsequently, the patient underwent laparotomy with a midline incision under general anesthesia. During exploration, the spleen was seen on the right side of the abdomen and measured 12 x 14 x 9 cm in maximum dimensions. The spleen appeared viable with no evidence of torsion or gangrene. The spleen was placed at its anatomical position and fixed with absorbable mesh. The operation was uncomplicated. The patient tolerated the procedure and had an uneventful recovery. The patient was discharged on the seventh postoperative day in good condition. He required this length of stay as the patient could not fully tolerate oral intake before the fifth postoperative day due to nausea.

## Discussion

We presented a rare case of wandering spleen in a middle-aged man who presented with a history of intermittent abdominal pain. Wandering spleen, also known as the hypermobile spleen, refers to the abnormal increase in mobility of the spleen so that it migrates from its normal position in the left hypochondriac fossa because of excessively long splenic pedicles and improper fixation of the splenic tissue to the underlying posterior abdominal wall [4,5]. The first case of wandering spleen was reported by Von Horne in 1667 [4]. It is reported that less than 600 cases of wandering spleen have been reported to date [6].

The wandering spleen may occur due to congenital or acquired etiologies. The congenital form involves the absent formation of the suspensory ligaments of the spleen. The spleen forms from the dorsal mesogastrium, which then rotates to the left side. The absence of suspensory ligaments results in long splenic pedicles, which are responsible for the increased mobility of the spleen. Furthermore, hypermobile colon and prune-belly syndrome are classic causes of the congenital wandering spleen. The acquired form of the wandering spleen has the same pathology of abnormal long splenic pedicles. The acquired form typically occurs in women of reproductive age, due to maternal hormones. It was found that both estrogen and progesterone increased the relaxin receptors on cells, promoting their action of increased ligamentous laxity [7]. Therefore, multiparous women are more prone to develop acquired wandering spleen. Nearly one-third of patients with wandering spleen present during childhood. Prepuberty and wandering spleen have equal gender preponderance. In adults, the prevalence of wandering spleen is much higher among women [1,3]. In the present case, the diagnosis of wandering spleen was unusual since the patient was male and had no identifiable risk factors [5].

The clinical manifestations of the wandering spleen are variable. The wandering spleen can be asymptomatic and found incidentally on physical examination or radiological investigations. It may also present with acute abdominal pain due to a splenic infarct. Furthermore, as in our case, the wandering spleen may present with intermittent abdominal pain that may be related to intermittent torsion and spontaneous detorsion of the splenic pedicle. Interestingly, our patient was clinically misdiagnosed as having gastritis and was on proton pump therapy for years. This highlights the importance of doing proper endoscopic and radiological investigations in patients with vague abdominal pain and not using an empiric treatment for common conditions without appropriate investigations. Acute presentations of the wandering spleen are likely due to torsion or mass effects on adjacent structures, causing intestinal obstruction, gastric volvulus, or pancreatitis [5]. The differential diagnosis of the wandering spleen depends on its presentation. For example, the differential diagnosis in patients with acute torsion includes ovarian torsion, acute appendicitis, and intestinal obstruction [5]. The presence of long splenic pedicles, which carry the blood supply to the spleen, predisposes to torsion of the pedicle leading to a partial or complete splenic infraction. Furthermore, the risk of torsion is aggravated if the spleen weight is greater than 500 grams [2].

The clinical diagnosis of a wandering spleen can be challenging, but the diagnosis can readily be made by cross-sectional imaging investigations. A computed tomography scan of the abdomen can demonstrate the absence of a spleen in its normal position in the left upper quadrant of the abdomen along with the presence of a mass in the lower abdominopelvic cavity that has similar attenuation to that of the normal splenic tissue. It can confirm the diagnosis and also identify the potential complications (e.g. splenic infarct). Ultrasound is another modality to diagnose the wandering spleen, as it is an affordable investigation and has no association with radiation risks. However, it is operator-dependent and its diagnostic accuracy is degraded by bowel gases, which may obscure the findings. In the present case, the patient underwent a computed tomography scan for a presumed diagnosis of perforated viscus given the findings of the clinical examination.

Surgical treatment is the only recommended management for wandering spleen [4]. Splenectomy is often required in cases of splenic torsion with infarction [3]. It may also be advised in the massive wandering spleen that cannot have splenopexy [4]. In other situations, splenic preservation with splenopexy should be attempted to avoid the future risks of overwhelming post-splenectomy sepsis [1]. Surgical treatment is needed even in asymptomatic patients because the rate of complications is substantial [4,5]. Laparoscopic splenopexy may also be used currently [8]. Conservative management is not advised, as it is associated with a complication rate that exceeds 50% [6].

## Conclusions

A wandering spleen is a rare medical condition that poses a clinical diagnostic challenge and requires a high index of suspicion. Cross-sectional imaging studies can readily confirm the diagnosis and identify the possible complications. Surgical treatment, preferably with splenopexy, should be performed even in mildly symptomatic patients to prevent the complication of torsion and infarction. The diagnosis of the wandering spleen should be considered in patients with recurrent abdominal pain.
